# Publication trends and hotspots of colorectal adenoma during 2002-2022: a bibliometric and visualized analysis

**DOI:** 10.3389/fonc.2023.1142703

**Published:** 2023-07-10

**Authors:** Xue Li, Wenzheng Zhang, Surui Yuan, Qiyuan Mao, Chuchu Zhang, Ruijuan Cai, Hongsheng Lin, Xueqian Wang

**Affiliations:** ^1^ Department of Oncology, Guang’anmen Hospital, China Academy of Chinese Medical Sciences, Beijing, China; ^2^ Graduate School, Beijing University of Chinese Medicine, Beijing, China; ^3^ Institution of Information on Traditional Chinese Medicine, China Academy of Chinese Medical Sciences, Beijing, China

**Keywords:** colorectal adenoma, colorectal cancer, bibliometric analysis, Research trends, hotspots

## Abstract

**Background:**

Prevention and treatment of colorectal adenoma (CRA) are great significant to reduce morbidity and mortality of colorectal cancer. Although there have been numerous studies on CRA recently, few publications utilized the bibliometrics to evaluate this field. The objective of current study was to provide a comprehensive analysis of the current state and frontier progress of CRA over the past 20 years.

**Methods:**

The Web of Science Core Collection was utilized to extracted all studies of CRA during 2002-2022. Bibliometric tools including CiteSpace, VOSviewer, and the Online Analysis Platform of Literature Metrology were used for statistical analysis. CiteSpace and the Online Analysis Platform were used to evaluate the contributions of various countries/regions, institutions, authors, and journals in this field. Research hotspots and trends were identified through keywords and references analysis by VOSviewer and CiteSpace.

**Results:**

2,268 publications from 2002 to 2022 in total were identified. The number of global publications in this field has increased annually. The USA was the most productive country, contributing nearly 30% of global publications. But in recent years, China’s publications grew rapidly and had the highest citation strength. The most productive institutions was the National Cancer Institute. Baron JA from the USA was the most productive and the one of most co-cited authors. *Cancer Epidemiology Biomarkers & Prevention* had the highest number of publications and *Gastroenterology* was the most co-cited journals. Analysis of keywords clusters showed that “mechanism/pathophysiology”, “risk factors and prevention”, “colonoscopy screening and treatment”, “metabolism”, and “microbiota” were the major frontier topics and the main research directions.

**Conclusions:**

CRA publications have shown a gradual upward trend in recent years, most of which have been published by developed countries. Developing countries should further focus on CRA research and transnational cooperation with developed countries in the future, in order to better improve the situation of the increasing morbidity and mortality of CRC. Baron JA was the most outstanding researcher in this field. More attention should be devoted to “pathogenesis of CRA”, “less invasive diagnostic methods”, “chemoprevention”, and “screening and risk prediction of CRA including gut microbiome and metabolism”, which will be frontiers in the future.

## 1 Introduction

Colorectal cancer (CRC) is one of the most common cancers with the highest fatality rate worldwide, where there were about 1.9 million new cases and 935,00 new deaths, respectively according to GLOBOCAN 2020 (1). Although CRC was considered to be a disease mainly of developed countries, quick rises in morbidity are happening in countries going through economic progress and mutations in diet and lifestyle (2). In developed countries, screening and revised treatment was reducing morbidity and mortality considerably (3). While in developing countries, existing screening programs and available medical treatment were currently deficient to curb the escalating rise in these rates (4). Thus, it is critically important to understand the prevention strategies of CRC. Studies showed that about 90% of CRC cases developed from colorectal adenoma (CRA), so CRA was considered to be one of the most important precancerous lesions of CRC. Older age was correlated with the incidence of CRA, studies showed that CRA were estimated to be present in 20 to 53% of the U.S. population older than 50 years of age (5, 6). Moreover, a study involving 157,943 Chinese persons who underwent colonoscopy between 1990 and 2009 showed that 6,777 patients had advanced CRA, and the detection rate in population older than 50 years (5,021,6.02%) was significantly higher than in population younger than 50 years(1,756,2.35%) (7). To sum up, because prevention of CRA can reduce mortality of CRC, it is imperative to conduct CRA-related research.

Endoscopic detection and removal of CRA are the main methods of diagnosis and treatment respectively, which could reduce the risk of CRC incidence and mortality (6, 8). Nevertheless, the recurrence of adenoma was high after polypectomy, which raises a big challenge for the treatment and prevention of CRA. A meta-analysis showed that the adenoma recurrence was 37% at one year, 41% at three years, and 60% at five years (9). Current guidelines recommended a 5-year or even 10-year surveillance interval for low risk of CRA after polypectomy, while a 1–3 year interval surveillance for high risk of CRA after polypectomy (10). In addition, repeated colonoscopy after polypectomy brought psychological and economic burdens to patients (11). Studies showed that the incidence and recurrence could be reduced by chemoprevention agents and improving lifestyle, but they were not gained general acceptance with adverse drug reactions and unsatisfied effectiveness (12, 13). To better solve these questions, it is important to understand most research and build a multidimensional research network through longitudinal and global perspectives to identify the hotspots and trends in CRA.

Bibliometrics, based on mathematics and statistics, is one way to analyze massive heterogeneous literature. So far, there has been no systematic global research trends in CRA, so it is necessary to investigate the entire position of CRA study to help scholars in tracking research development. The present research problem is that the diagnosis and treatment of CRA are in a period of quick transformation; however, there is a research gap in the lack of scientific analysis to sum up this trend. In case of a scientific study is conducted by bibliometrics, it will promote scholars’ comprehending of the CRA research fields and increase the efficiency of scientific research (14, 15). Hence, we aimed to determine the countries, institutions, authors, and journals with the publications of CRA. And through comprehensive and objective analysis of reference data, we summarized the current hotspots and frontiers of CRA for future research.

## 2 Methods

### 2.1 Data source and search strategies

**Figure 1:**
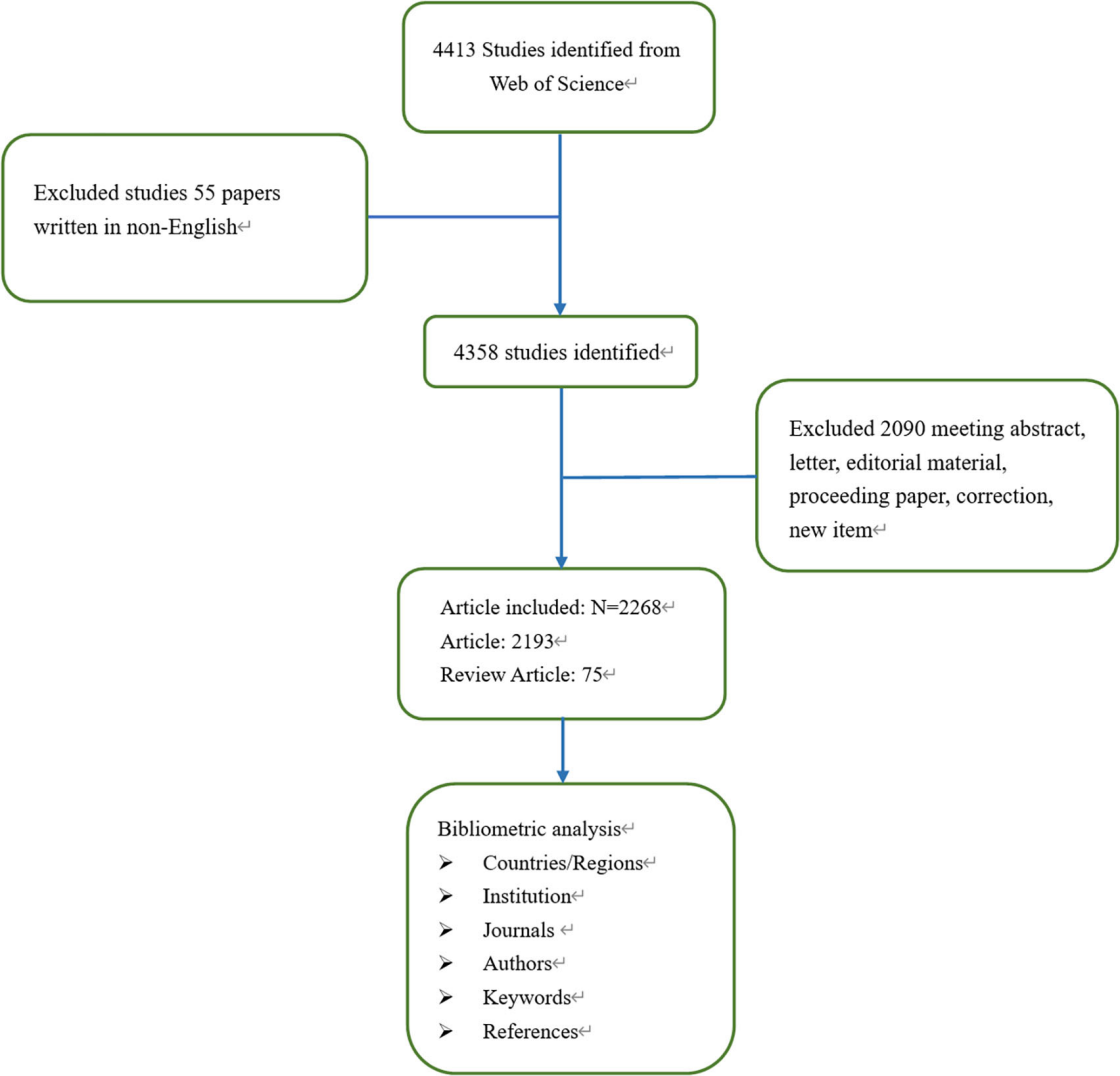
Flow chart of the searching strategy of the data selection process.

The Web of Science Core Collection (WoSCC) was selected to conduct a bibliometric analysis for the following reasons (1): WoSCC is the most authoritative, comprehensive, and widely used global database; (2) WoSCC provided the detailed literature information needed for software including CiteSpace, VOSviewer, and Biblioshiny; (3) Our choice of WoSCC was also based on previous studies and researches of Chaomei Chen, the developer of CiteSpace (16–18). Therefore, WoSCC database was chosen as the data source. The search strategy employed was as follows: TI=(colorectal OR colorectum OR colon OR rectum OR bowel) AND TI=(adenomatous OR adenoma). The inclusion criteria included: (1) The time span was from 2002 to 2022; (2) document language was limited to English; (3) document types including articles and reviews. The exclusion criteria included: (1) irrelevant meeting abstract, letter, editorial material, proceeding paper, corrected, and new item; (2) unpublished documents without enough information for further analysis. To reduce bias incurred by database updating, all data retrieval and collection were finished on May 29, 2022. The data processing flowchart was shown in **Figure 1**.

### 2.2 Data collection

Two researchers (Xue Li and Xueqian Wang) independently screened the abstracts individually and reached a consensus on the qualifying papers. Data including the titles, countries/regions, institutions, journals, authors, and keywords was subsequently exported and saved as TXT. format. CiteSpace software 5.7.R3 (Drexel University, Philadelphia, PA, USA), VOSviewer software 1.6.18 (Leiden University, Netherlands), Biblioshiny software, and the Online Analysis Platform of Literature Metrology (http://bibliometric.com/) were employed to construct visualized maps of scientific literature.

### 2.3 Bibliometric analysis

All publication characteristics were well recorded and described. We got access to the impact factors (IF) of the relevant journals by the current edition of JCR (Journal Citation Reports), which is a basic criterion for the assessment of academic influences. The H-index acquired from WoSCC has been widely accepted for evaluating the scientific contribution, which is defined as the number of papers with citation number ≥h (the ranking position where the number of publications is greater than the number of citations). The number of annual publications and growth trends were analyzed by the Online Analysis Platform of Literature Metrology and Microsoft Excel.

CiteSpace V5.8 R3 was mainly employed to create citation analysis mapping, co-citation analysis mapping, co-authorship analysis mapping, citation bursts mapping, journal dual-map, and keyword timeline mapping, most of which consisted of nodes and links, with different meanings in different analysis methods. In citation/co-citation/co-authorship analysis, the nodes represented an item, and the size represented frequency. Moreover, the line between the two nodes represented the relationship between the research items; darker color and thicker line represented the greater cooperation intensity between nodes. The centrality was an index, which could reflect the significance of nodes in the cooperation networks. Nodes with a centrality value of more than 0.1 occupied the pivotal locations connecting nodes, and were regarded as central nodes displayed in purple rings. Bursts detection could reflect emerging academic trends and new topics, predicted frontier research directions, and revealed potential hotspots in a field; the blue line was timeline, whereas citation bursts detection was shown as a red segment on the blue timeline, which indicated the start year, end year, and duration of the bursts. Z-score and F-score were used to re-adjust or standardize the citation data and could be used to identify major citation paths in the dual-map. The details on CiteSpace settings were as follows: time span(2002-2022), years slices (1 or 3), pruning (Minimum Spanning Tree and Pruning Sliced Networks), and selection criteria (Top N=50). Other parameters were set to the default settings.

VOSviewer 1.6.18 was mainly conducted keyword cluster analysis in this study. The nodes and lines in the VOSviewer maps stood for the weights and associations of the study objectives, where the scientific mapping generated by the VOSviewer automatically groups the nodes into different colors by clusters. The settings of the VOSviewer were as follows: Created a map based on bibliographic data (WOS files), Type of analysis (co-occurrence), Unit of analysis (keywords), Chose threshold (filter according to the output results, Keywords: 10 co-occurrences).

Biblioshiny software, bibliometrix package in R version 4.3.0 was performed to visualize a three-filed plot to show the relationship between authors, institutes, countries, and keywords. In addition, Biblioshiny software was also conducted a thematic map to show the classification based on keywords.

## 3 Results

**Figure 2:**
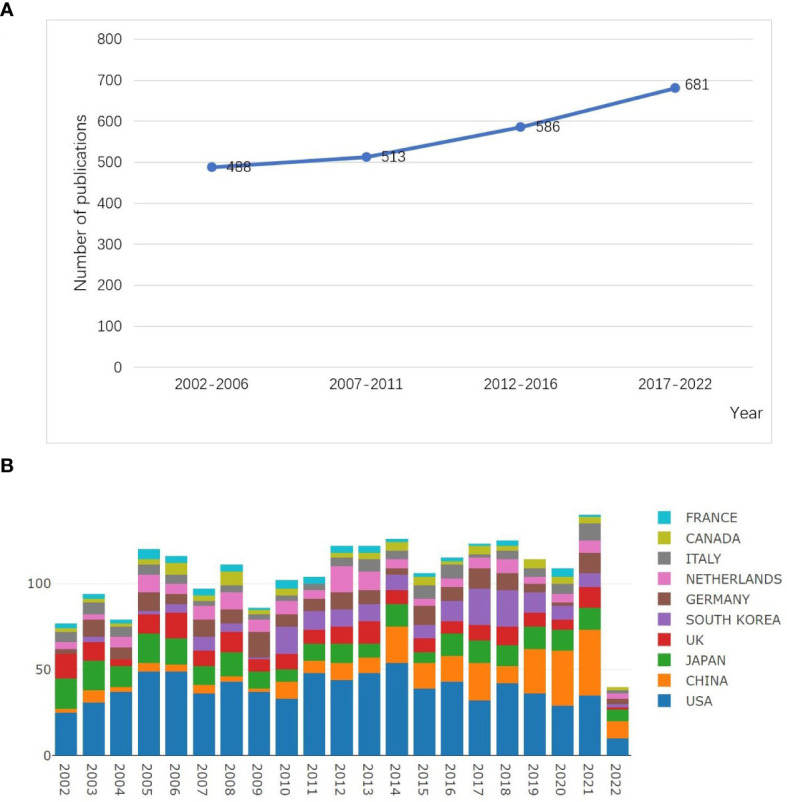
Output of related publications in the research of CRA. **(A)** The number of publications and growth trends every five years. **(B)** The number of annual publications and growth trends of the top 10 countries/regions. Source: authors with the Online Analysis Platform of Literature Metrology (http://bibliometric.com/).

A total of 4,413 publications were extracted, and 2,268 publications were finally confirmed, including 2,193(97%) original articles and 75(3%) reviews. The total number of citations for the retrieved articles was 87,331, and the mean citations per article was 35. The H-index was 115. The volume of publications related to CRA in the past 20 years was shown in **Figure 2A**. In general, the number of publications in the past ten years has increased compared with the previous ten years with the greatest growth occurring in the recent five years.

### 3.1 Analysis of countries/regions

**Figure 3:**
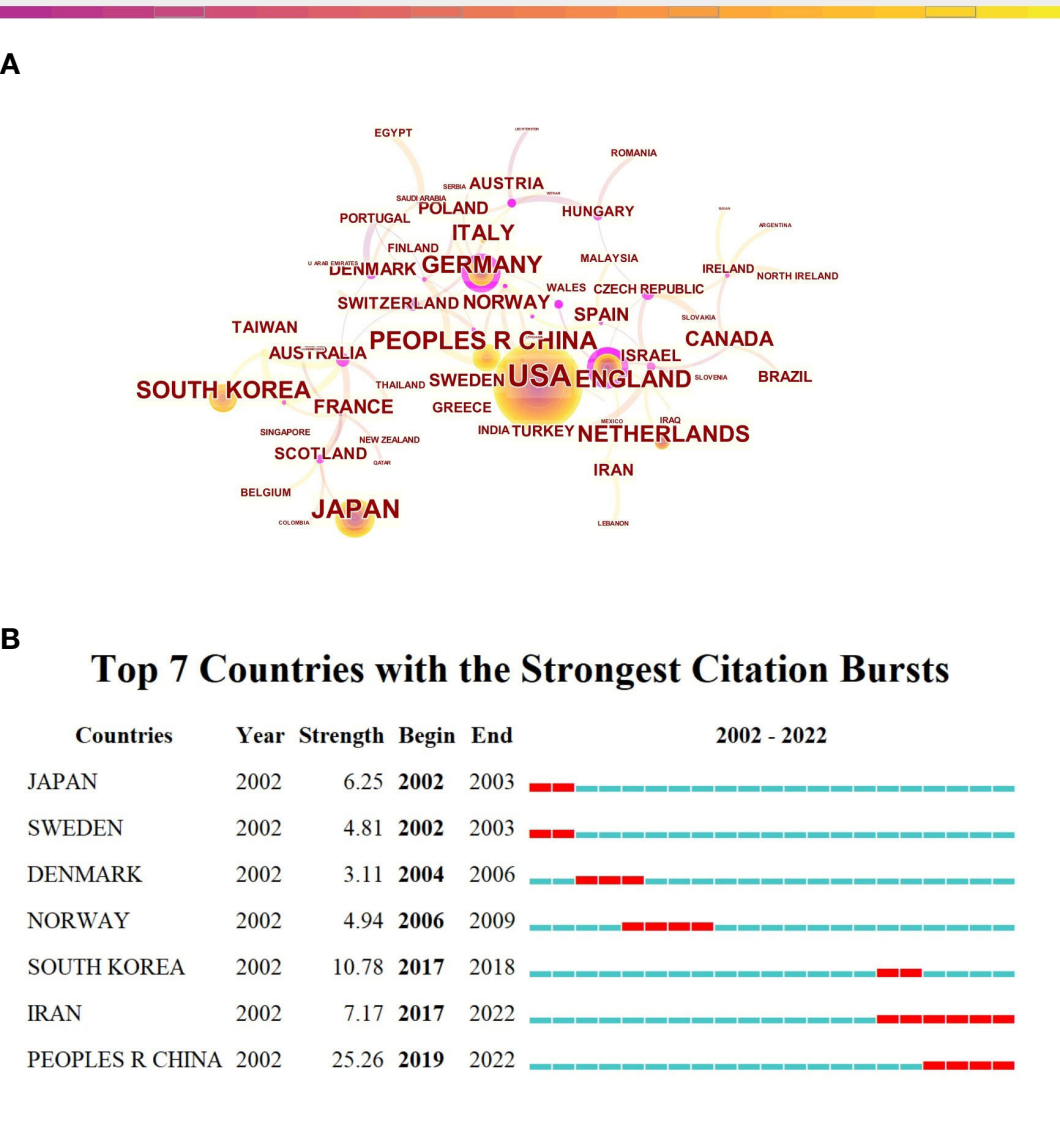
Countries’ scientific productions and collaboration relationship in the research of CRA. **(A)** Collaboration network of countries. A node represented a country, the larger the node means more publications. A line represented the collaboration relationship between two countries, darker color and thicker line indicated the greater cooperation intensity between nodes. The purple rings meant the node’s centrality ≥ 0.1, referred to the central node in the cooperation network. **(B)** The top 7 countries/regions with the strongest citation bursts. The red segment of the blue line was citation bursts detection, which indicated the start year, end year, and duration of the bursts.

A total of 63 countries/regions performed research in this field. CiteSpace software was conducted to analyze the research of each country, and the top 10 most productive countries/regions were shown in **Table 1**. In **Figure 3A**, all countries appeared 2,845 times in total, among which the USA had the most publications (799,28.08%), followed by Japan (251,8.82%), and China (225,7.91%). However, the top 3 countries with highest centrality were Australia (centrality=0.48), Finland(0.57), and England (0.46). In terms of citation frequency of literature, the top 3 countries were the USA (43,025 times), Japan(8,875 times), and China (6,505 times). Although China ranked third in the number of publications, the H-index of China was 41, which was in sixth place. **Figure 2B** illustrated the dynamics of the number of publications and growth trends in the top 10 countries through the Online Analysis Platform of Literature Metrology. Although China was initially lagging behind, its annual publication output in this field grew rapidly, indicating its intense vitality of research in this field. Besides, we analyzed the top 7 countries with the strongest citation bursts conducted by CiteSpace, as shown in **Figure 3B**, China had the highest citation strength. Meanwhile, China and Iran performed well in the citations of papers recently. These phenomenon all reflected that China had a quite degree of influence in the recent four years.

**Table 1:** The top 10 countries/regions contributing to publications in the research of CRA during 2002 to 2022 (sorted by count).

Ranking	Country	Count	Percentage (N/2845)	Centrality	Total citations	H-index
1^st^	USA	799	28.08	0.06	43025	88
2^nd^	Japan	251	8.82	0.00	8875	46
3^rd^	China	225	7.91	0.00	6505	41
4^th^	England	170	5.98	0.46	11886	47
5^th^	Germany	169	5.94	0.00	6039	45
6^th^	South Korea	165	5.80	0.06	3622	32
7^th^	Netherlands	137	4.82	0.07	7958	43
8^th^	Italy	110	3.87	0.12	3847	31
9^th^	Canada	76	2.67	0.14	7831	32
10^th^	France	59	2.07	0.18	3109	28

### 3.2 Analysis of institutions

**Figure 4:**
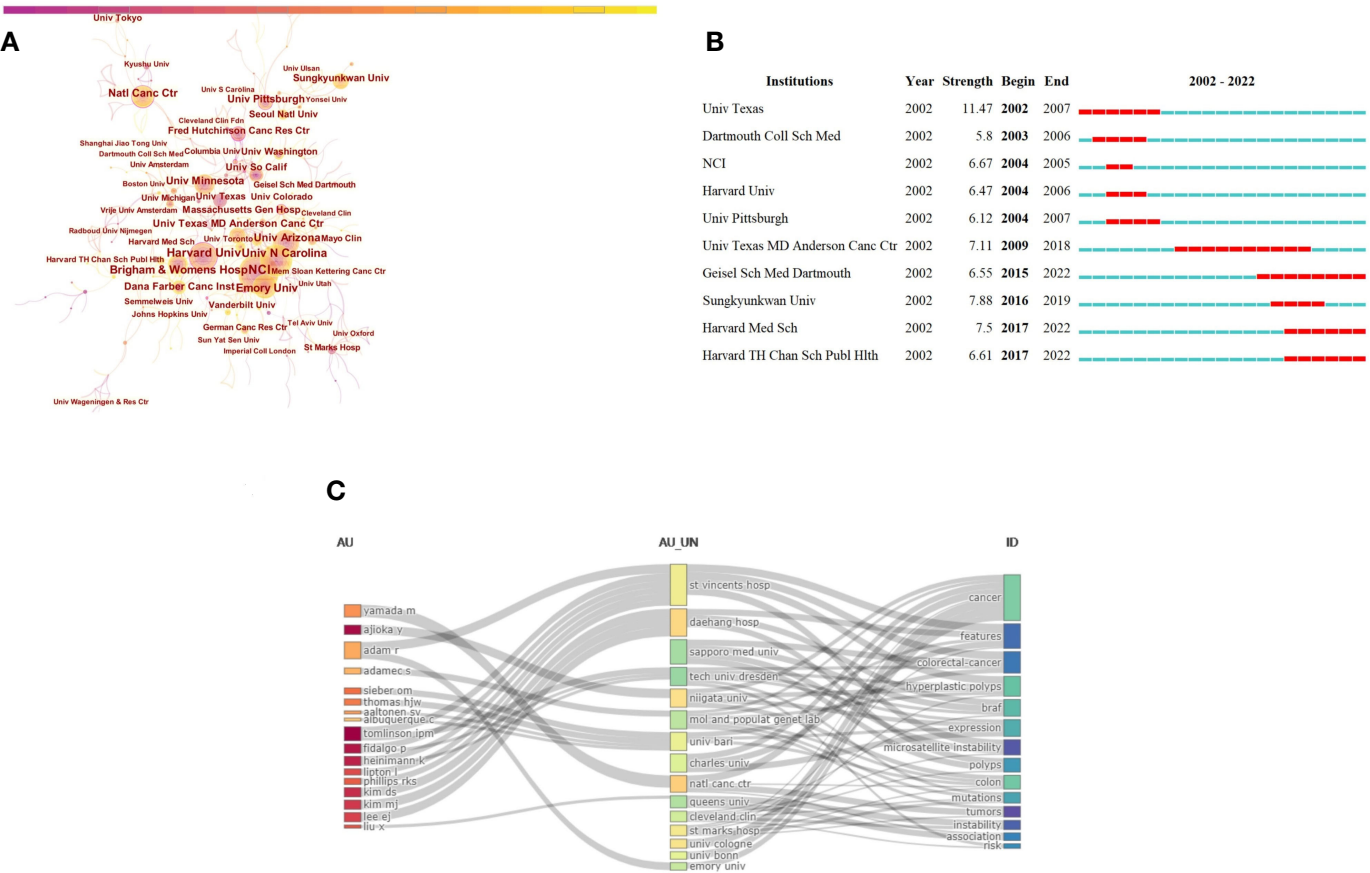
Visualization of institutions analysis in the research of CRA. **(A)** Collaboration network of institutions. A node represented a institution, the larger the node means more publications. A line represented the collaboration relationship between two instituions, darker color and thicker line indicated the greater cooperation intensity between nodes. The purple rings meant the node’s centrality ≥ 0.1, referred to the central node in the cooperation network. **(B)** The top 10 institutions with the strongest citation bursts. The red segment of the blue line was citation bursts detection, which indicated the start year, end year, and duration of the bursts. **(C)** A three-field plot showing the network between authors (left), institutions (middle), keywords(right).

The results of the collaborative relationship between different institutions showed 582 nodes and 643 links (**Figure 4A**). National Cancer Institute (NCI) (105,18.04%) was the most productive institution, followed by Harvard University (74,12.71%) and University of North Carolina at Chapel Hill (74,12.71%) (**Table 2**). Among these 10 institutions, the centrality of Harvard University (centrality=0.14), National Cancer Center Japan (0.11), University of Minnesota (0.10), and University of Pittsburgh (0.11) was greater than 0.1, which indicated that the influence degree and cooperation degree of these four institutions were high in recent 20 years. Noteworthily, of the top 10 institution with respect to the number of articles published, nine were from the USA, and remaining one was the National Cancer Center Japan. Despite having only 23 publications, Vanderbilt University ranked first in the world with a centrality of 0.16. Moreover, we analyzed the top 10 institutions with the strongest citation bursts, University of Texas System ranked first with a citation strength of 11.47. However, Geisel School of Medicine at Dartmouth, Harvard Medical School, and Harvard T.H. Chan School of Public Health performed well in the citations of papers recently (**Figure 4B**).

**Table 2:** The top 10 institutions contributing to publications in the research of CRA during 2002 to 2022 (sorted by count).

Ranking	Institutions	Country	Count	Percentage (N/582)	Centrality
1^st^	NCI	USA	105	18.04	0.06
2^nd^	Harvard University	USA	74	12.71	0.14
3^rd^	University of North Carolina at Chapel Hill	USA	74	12.71	0.05
4^th^	Brigham and Women’s Hospital	USA	62	10.65	0.06
5^th^	Emory University	USA	61	10.48	0.03
6^th^	University of Arizona	USA	54	9.28	0.06
7^th^	National Cancer Center Japan	Japan	52	8.93	0.11
8^th^	University of Minnesota	USA	47	8.08	0.10
9^th^	University of Pittsburgh	USA	41	7.04	0.11
10^th^	University of Texas MD Anderson Cancer Center	USA	40	6.87	0.03

A three-filed plot that showed the relationship between institutions, authors, and keywords was displayed in **Figure 4C**. The height of nodes was proportional to the number of occurrence of a certain author, institution, and keyword within the network. The width of the gray lines between the rectangle nodes was proportional to the frequency of connections. The figure indicated that St Mark’s Hospital, UK (n=31) was the institution with the most connections between authors and keywords, followed by Daehang Hospital, Korea (n=18), and Sapporo Medical University, Japan (n=16).

### 3.3 Analysis of journals

**Figure 5:**
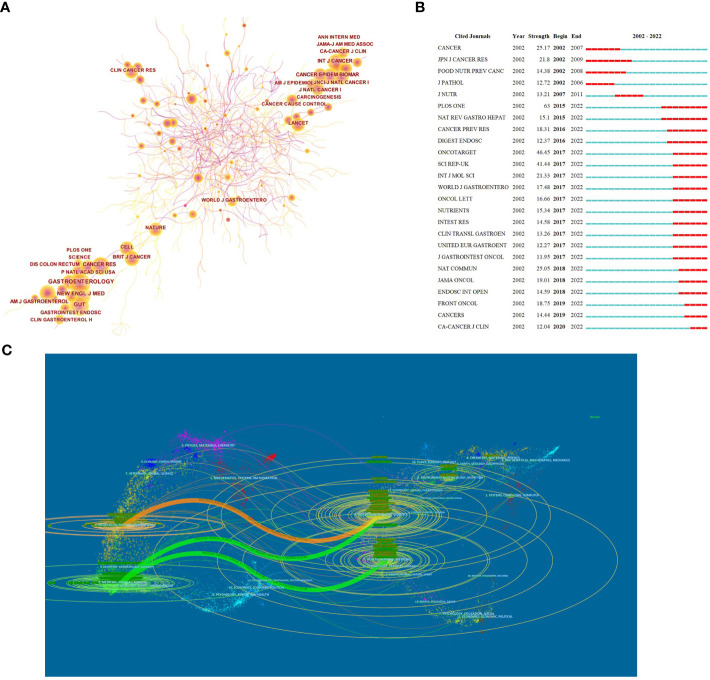
Published journals in the research of CRA. **(A)** Collaboration network of journals. A node represented a institution, the larger the node means more publications. A line represented the collaboration relationship between two instituions, darker color and thicker line indicated the greater cooperation intensity between nodes. The purple rings meant the node’s centrality ≥ 0.1, referred to the central node in the cooperation network. **(B)** The top 25 journals with the strongest citation bursts. The red segment of the blue line was citation bursts detection, which indicated the start year, end year, and duration of the bursts. **(C)** A dual-map overlap of journals. The citing journal were at left, the cited journals were on the right. The colored path represented the cited relationship.

In total, 537 academic journals have published papers about CRA. **Table 3** showed the top 10 productive journals, which have published 518 publications, accounting for 23% of the total publications. In cited journals analysis, *Cancer Epidemiology Biomarkers & Prevention* (n=107) ranked first, followed by *International Journal of Cancer*(n=59), *Cancer Prevention Research*(n=54), *Gastrointestinal Endoscopy*(n=50); And six had an Impact Factor(IF) of more than five. Among the top 10 most productive journals, *Gut* had the highest IF of 23.06. In addition, the co-citation analysis of journals was presented as a network map with 792 nodes and 7,909 links (**Figure 5A**). The results showed that the most frequently co-cited journals were *Gastroenterology*(n=1474), followed by *The New England Journal of Medicine*(n=1,211), *and Gut*(n=1,134) (**Table 4**); And nine were at Q1 JCR division with an IF of more than seven. Meanwhile, more and more publications performed well in the number of citations in the recent years (**Figure 5B**).

**Table 3:** Top 10 most active journals contributing to publications in the research of CRA during 2002 to 2022(sorted by count).

Ranking	Journal	Count	IF(2020)^#^	JCR	H-index
1st	Cancer Epidemiology Biomarkers & Prevention	107	4.25	Q2	36
2^nd^	International Journal of Cancer	59	7.40	Q1	29
3^rd^	Cancer Prevention Research	54	3.49	Q3	20
4^th^	Gastrointestinal Endoscopy	50	9.43	Q1	23
5^th^	Gut	45	23.06	Q1	29
6^th^	World Journal of Gastroenterology	45	5.74	Q2	17
7^th^	Plos One	45	3.24	Q2	17
8^th^	Digestive Diseases and Sciences	39	3.20	Q3	13
9^th^	Gastroenterology	37	22.68	Q1	28
10th	Cancer Causes Control	37	2.51	Q4	19

**Table 4:** Top 10 most active co-cited journals contributing to publications in the research of CRA during 2002 to 2022 (sorted by count).

Ranking	Co-cited Journal	Count	Centrality	IF(2020)^#^	JCR	H-index
1st	Gastroenterology	1474	0.07	22.68	Q1	28
2^nd^	The New England Journal of Medicine	1211	0.00	91.25	Q1	12
3^rd^	Gut	1134	0.00	23.06	Q1	29
4^th^	Cancer Research	1065	0.09	12.70	Q1	25
5^th^	International Journal of Cancer	1014	0.00	7.40	Q1	29
6^th^	Cancer Epidemiology, Biomarkers & Prevention	865	0.03	4.25	Q2	36
7^th^	The American Journal of Gastroenterology	864	0.02	10.86	Q1	24
8^th^	British Journal of Cancer	659	0.02	7.64	Q1	21
9^th^	Proceedings of the National Academy of Sciences of the United States of America	622	0.01	11.21	Q1	7
10th	JNCI-Journal of the National Cancer Institute	505	0.00	13.51	Q1	8

The dual-map overlay could well show the distribution of journals and the relationship between journals and cited journals (the color path represented the cited relationship). **Figure 5C** identified four main reference paths. It could be seen that there were mainly four citation paths, and the citing papers were mainly concentrated in three fields: (1)Molecular, Biology and Immunology →Molecular, Biology, Genetics (z=4.75,f=12,826); (2) Medicine, Medical, Clinical →Molecular, Biology, Genetics (z=4.58,f=12,388); (3) Medicine, Medical, Clinical →Health, Nursing, Medicine (z=3.69,f=10,118).

### 3.4 Analysis of authors

**Figure 6:**
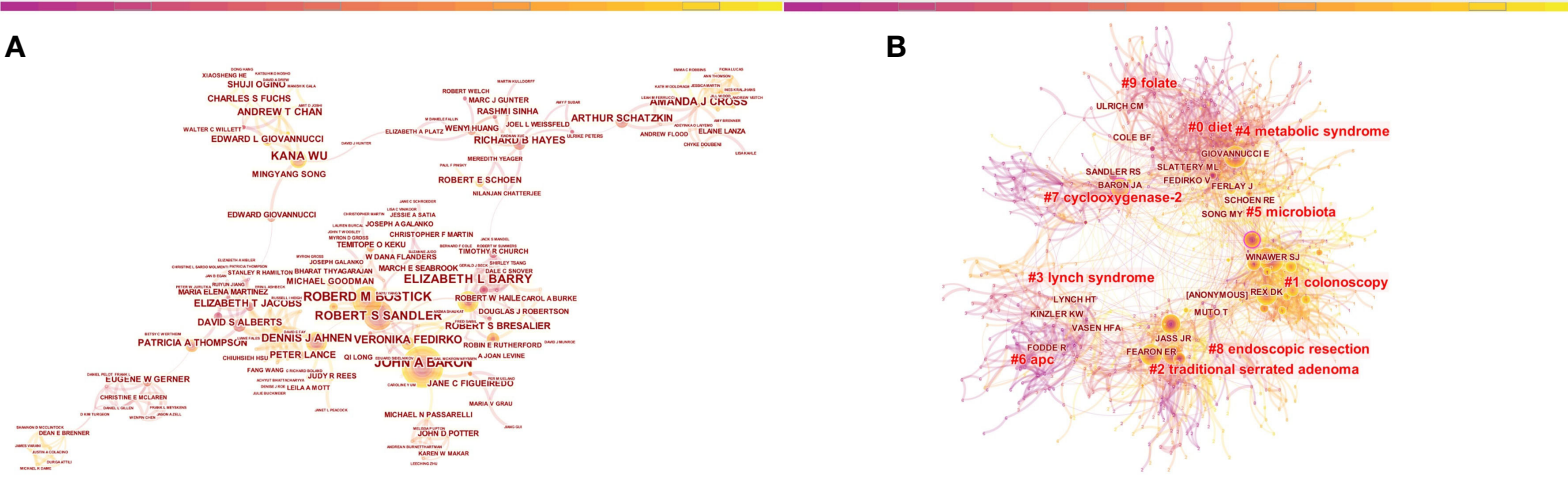
Visualization of authors analysis in the research of CRA. **(A)** Collaboration network of authors. A node represented an author, the larger the node means more publications. A line represented the collaboration relationship between two authors, darker color and thicker line indicated the greater cooperation intensity between nodes. **(B)** The mixed visualization map of both co-cited author and clusters of keywords.

In most cases, numerous researchers are needed to cooperate on a study, and their contributions are described as a ranking of authors. We could assess the key authors and their collaboration in a research field by analyzing the characteristics of authors’ cooperative networks through CiteSpace. In co-authorship analysis, results included 742 nodes and 1838 links (**Figure 6A**). The most productive authors were Baron JA (n=40), followed by Bostick RM (n=39) and Sandler RS (n=32) (**Table 5**). Baron JA, a professor at University of North Carolina at Chapel Hill, was ranked second in analysis of the co-cited author. At the same time, 4 of the top 10 most cited papers were published by him (**Table 6**), which showed that he has made outstanding achievements in the research fields of CRA. However, the centrality of the top 10 authors was not high. Of note, although Professor Sandler RS from University of North Carolina at Chapel Hill only ranked third in the number of publications, he ranked first with 7760 citations and a H-index of 29. As shown in **Table 7**, the most co-cited authors were Winawer SJ(n=348), followed by Giovannucci EL(n=327), Rex DK(n=323), and Fearon ER(n=295), suggesting that their works might serve as a bridge between various studies. In general, most of these nineteen influential authors came from the USA, and the other three came from the UK and Germany, which was slight difference in the result of the high productive countries, neither Japanese nor Chinese authors made the list. Moreover, the mixed visualization map of both co-cited authors and keywords was shown in **Figure 6B**. Each node represented an author, and each keyword represented a cluster. In the cluster of co-cited authors, the presentative cluster labels included diet, colonoscopy, traditional serrated adenoma, lynch syndrome, metabolic syndrome, microbiota, APC, cyclooxygenase-2, endoscopic resection and folate.

**Table 5:** The top 10 most productive authors contributing to publications in the research of CRA during 2002 to 2022 (sorted by count).

Ranking	Author	Country	Count	Centrality	Total citations	H-index
1st	Baron JA	USA	40	0.00	6849	27
2^nd^	Bostick RM	USA	39	0.00	1489	22
3^rd^	Sandler RS	USA	32	0.01	7760	29
4^th^	Barry EL	USA	29	0.01	1390	14
5^th^	Wu K	USA	25	0.01	819	13
6^th^	Fedirko V	USA	23	0.00	462	12
7^th^	Cross AJ	UK	23	0.01	976	16
8^th^	Ahnen DJ	USA	22	0.02	1989	19
9^th^	Chan AT	USA	16	0.00	1095	18
10th	Hayes RB	USA	16	0.00	1762	25

**Table 6:** The top 10 most frequently cited references in the research of CRA during 2002 to 2022.

Rank	Title	Corresponding authors	Journal	Year	Total citations	Average citations per year (rank)
1	Cardiovascular events associated with rofecoxib in a colorectal adenoma chemoprevention trial	Bresalier RS	The New England Journal of Medicine	2005	1882	104.56 (3)
2	Long-term expansion of epithelial organoids from human colon, adenoma, adenocarcinoma, and barrett’s epithelium	Sato T	Gastroenterology	2011	1776	148 (1)
3	Cardiovascular risk associated with celecoxib in a clinical trial for colorectal adenoma prevention	Solomon SD	The New England Journal of Medicine	2005	1591	88.39 (4)
4	A randomized trial of aspirin to prevent colorectal adenomas	Baron JA	The New England Journal of Medicine	2003	1078	53.9 (6)
5	Adenoma detection rate and risk of colorectal cancer and death	Corley DA	The New England Journal of Medicine	2014	1029	114 (2)
6	A randomized trial of aspirin to prevent colorectal adenomas in patients with previous colorectal cancer	Sandler RS	The New England Journal of Medicine	2003	857	43 (9)
7	Celecoxib for the prevention of sporadic colorectal adenomas	Bertagnolli MM	The New England Journal of Medicine	2006	799	47 (7)
8	Celecoxib for the prevention of colorectal adenomatous polyps	Arber N	The New England Journal of Medicine	2006	755	44 (8)
9	Folic acid for the prevention of colorectal adenomas - A randomized clinical trial	Cole BF	JAMA-The Journal of the American Medical Association	2007	663	41 (10)
10	Multiple colorectal adenomas, classic adenomatous polyposis, and germ-line mutations in MYH	Sieber OM	The New England Journal of Medicine	2003	629	63 (5)

**Table 7:** The top 10 most productive co-cited authors contributing to publications in the research of CRA during 2002 to 2022 (sorted by count).

Ranking	Co-cited author	Count	Country	Centrality	Total citations	H-index
1st	Winawer SJ	348	USA	0.02	2819	3
2^nd^	Giovannucci EL	327	USA	0.06	1246	21
3^rd^	Rex DK	323	USA	0.06	3515	10
4^th^	Fearon ER	295	USA	0.04	72	2
5^th^	Lieberman DA	283	USA	0.07	2973	7
6^th^	Martinez ME	233	USA	0.12	1012	17
7^th^	Jass JR	222	UK	0.05	605	5
8^th^	Baron JA	203	USA	0.12	6849	27
9^th^	Vogelstein B	199	USA	0.04	215	1
10th	Brenner H	167	German	0.02	1060	11

### 3.5 Analysis of keywords

**Figure 7:**
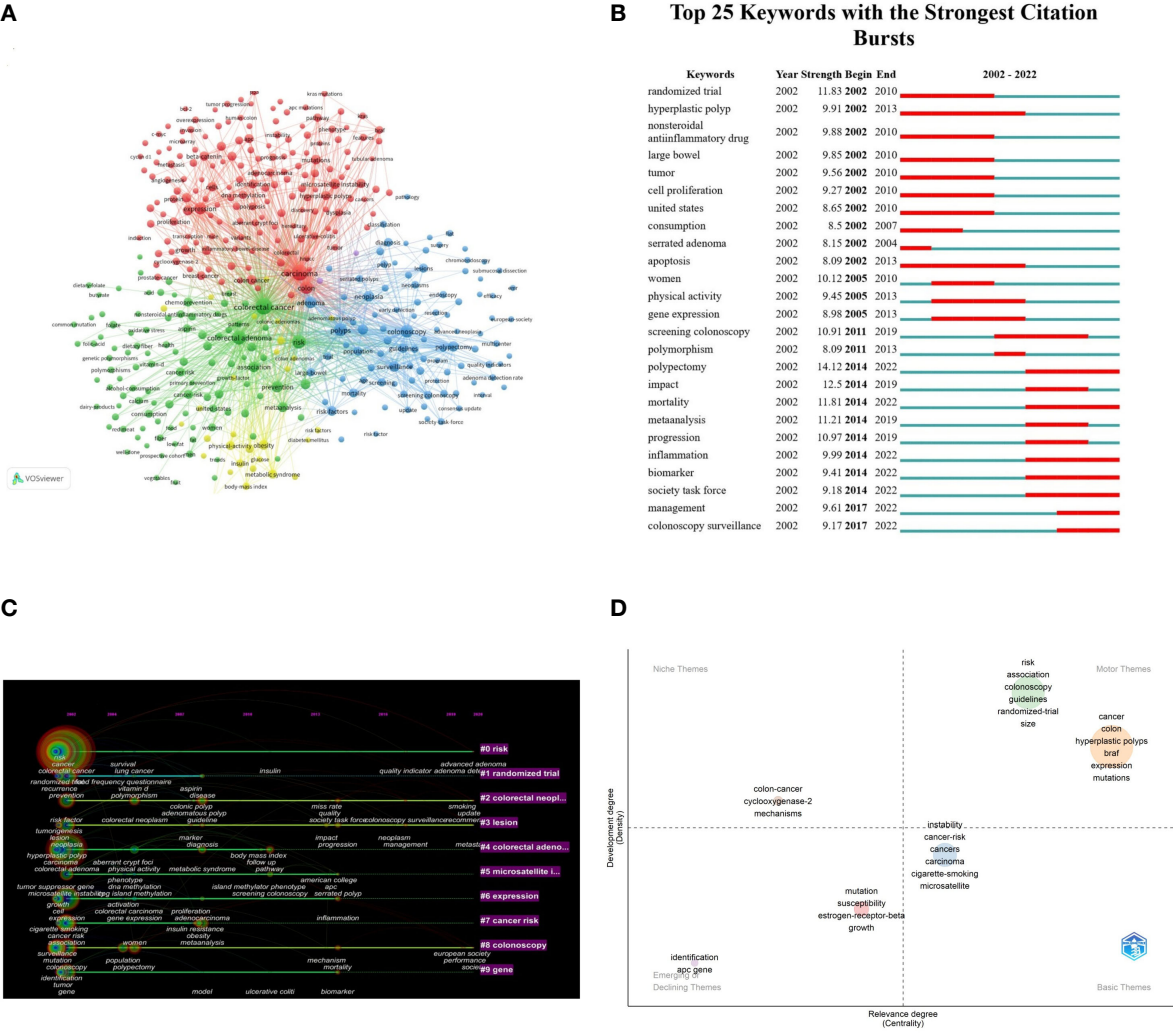
Visual mapping of keywords in the research of CRA. **(A)** Co-occurrence map of the network and clusters of keywords. The size of node and word reflects the co-occurrence frequencies, the link indicate the co-occurrence relationship, and the same color of node represent the same cluster. **(B)** The top 25 keywords with the strongest citation bursts. The red segment of the blue line was citation bursts detection, which indicated the start year, end year, and duration of the bursts. **(C)** The timeline view of keywords clusters. The label on the right indicated the clusters, the nodes indicated the keywords. Nodes with red tree rings refer to keywords with citation bursts. **(D)** A thematic map showing spots based on keywords classification. The X-axis represented the centrality indicating the importance of a keyword classification; The Y-axis symbolized the density indicating the development of a keyword classification.

Keywords reflected the core of the article, which could be used to analyze the frontiers of knowledge related to the research field. 6,483 keywords were extracted by VOSviewer. As shown in **Figure 7A**, the keywords mainly formed 5 clusters, representing the 5 major research directions and research scopes. The red cluster (cluster 1) focused on the research of mechanism/pathophysiology. The green cluster (cluster 2) focused on risk factors and prevention. The blue cluster (cluster 3) focused on colonoscopy screening and treatment. The yellow cluster (cluster 4) focused on metabolism. There was only two keyword (colorectal neoplasm and large bowel) in cluster pink (cluster#5).

Timeline view reflected the time of a topic in this field and showed this field’s evolutionary trajectory (**Figure 7C**). The position and size of the node on the timeline revealed the cumulative frequency, and the year for the first occurrence of each keyword, respectively. The solid line represented the duration of the hotspots. We found that risk, colorectal neoplasm, lesion, and colonoscopy had always been hot topics since 2002 until 2022. **Figure 7B** presented the top 25 references with the strongest citation bursts. Polypectomy (14.12) showed the strongest burst strength, followed by randomized trail (11.83) and mortality (11.81). In recent years (2014-2022), polypectomy, mortality, inflammation, biomarker, society task force, management, colonoscopy surveillance were the main research hotspots.

In **Figure 7D**, Biblioshiny software was contructed a thematic map to show spots based on keyword classifications with centrality as the X-axis and density as the Y-axis. In this figure, centrality referred to the relevance of a certain keyword classification to the CRA research field, and density indicated the degree of development of these keyword classifications. Two classifications in the upper right quadrant (motor theme) has high density and centrality, representing well-developed and important themes in the CRA research field. Cluster 1 included “risk”, “association”, “colonscopy”, “guidelines”, “randomized-trail”, and “size”. Cluster 2 included “cancer”, “colon”, “hyperplastic polyps”, “braf”, “expression”, and “mutations”. Only one classification in the left quadrant (niche theme) and the cluster included “colon-cancer”, “cyclooxygenase-2”, and “mechanism”. And there were two classifications in the emerging or declining theme: cluster 1 consisted of “indentification” and “apc gene”, and cluster 2 contained “mutation”, “susceptibility”, “estrogen-receptor-beta”, and “growth”. The basic theme contained one cluster, and the classification mainly included “instability”, “cancer-risk”, “cigarette-smoking”, and “microsatellite”. Based on the above result, we could include that these clusters by Biblioshiny coincide with the clusters of CiteSpace and Vosviewer to some extent.

### 3.6 Analysis of references

**Table 6** listed the top 10 high-cited references on CRA from 2002 to 2022. These articles had 11,059 citations, representing 15% of total citations. “Cardiovascular events associated with rofecoxib in a colorectal adenoma chemoprevention trial” was conducted by Bresalier RS et al. and published in *N Engl J Med* in 2005 (19). The total citation and average annual citation frequencies of this research were up to 1882 and 104.56, respectively. Regarding the top 10 high-cited references, *N Engl J Med* published eight articles, whereas the remaining two high-cited articles were published in *Gastroenterology* and *JAMA*, respectively. **Table 8** listed the top 10 co-cited references, of which Zauber AG, et al. with 67 co-citations (20), followed by Lieberman DA, et al. (21) and Baron JA, et al. (22) with 51 and 41 co-citations, respectively. Noteworthily, two references, Baron JA, 2003, *N Engl J Med* (22), and Corley DA, 2014, *N Engl J Med* (23), were both appeared in top 10 high-cited and co-cited references lists. In general, these publications’ academic values in this field were greatly accepted.

**Table 8:** The top 10 most frequently co-cited references in the research of CRA during 2002 to 2022.

Rank	Title	Corresponding authors	Journal	Year	Count	Centrality
1	Colonoscopic polypectomy and long-term prevention of colorectal-cancer deaths	Zauber AG	The New England Journal of Medicine	2012	67	0.04
2	Guidelines for colonoscopy surveillance after screening and polypectomy: a consensus update by the US Multi-Society Task Force on Colorectal Cancer	Lieberman DA	Gastroenterology	2012	51	0.03
3	A randomized trial of aspirin to prevent colorectal adenomas	Baron JA	The New England Journal of Medicine	2003	41	0.07
4	Adenoma detection rate and risk of colorectal cancer and death	Corley DA	The New England Journal of Medicine	2014	33	0.06
5	Comprehensive molecular characterization of human colon and rectal cancer	Muzny DM	Nature	2012	33	0.05
6	Global patterns and trends in colorectal cancer incidence and mortality	Arnold M	Gut	2017	30	0.05
7	Association of colonoscopy and death from colorectal cancer	Baxter NN	Annals of Internal Medicine	2009	29	0.13
8	Quality indicators for colonoscopy and the risk of interval cancer	Kaminski MF	The New England Journal of Medicine	2010	28	0.13
9	Once-only flexible sigmoidoscopy screening in prevention of colorectal cancer: a multicentre randomized controlled trial	Atkin WS	Lancet	2010	28	0.11
10	Serrated lesions of the colorectum: review and recommendations from an expert panel	Rex DK	The American Journal of Gastroenterology	2012	27	0.08

## 4 Discussion

A total of 2,268 publications with respect to CRA from 2002 to 2022 were obtained by WoSCC. From the perspective to the number of publications, the publications of CRA have gradually developed in the past 20 years (**Figure 2A**). China’s publications surged after 2014 and surpassed the USA for the first time in 2020, and has ranked first ever since every year. Although the number of publications from the USA was significantly higher than other countries, the annual growth rate of publications from the USA, and other countries including South Korea and the Netherlands, slowed down significantly in the past three years. By comparison, the Chinese researchers maintained interest and attention in this research field, and the annal publications also kept growing, which was closely related to attach importance to prevention of CRC and provided financial support by Chinese government. Moreover, to our knowledge, this is the first bibliometric analysis of CRA. In this paper, we determined the trends and hotspots in this field. The main discussions were as follows.

### 4.1 Contribution analysis of countries/regions, institutions, journals and authors

In the term of the volume of publications, the 10 leading countries included five European countries, three Asian countries, and two American countries. The top two productive countries were the USA and Japan, respectively. Although China ranked third, its academic influence was not as leading as the volume of publications. China ranked sixth with an H-index of 41. Besides, among the top 10 countries with the productive publications over the past two decades, nine were from developed countries. And in the centrality analysis, the top three countries with the most cooperation degree were Australia, Finland and the United Kingdom. These phenomenon in part explained the academic impact of developed countries. Research in the developed countries seemed to have an excellent environment and conditions, characterized by leading technology, professional scholars, sufficient capital, and active academic communication. Among them, the USA was the typical one, and these advantage well explained why the USA became a leading force in this field.

Overall, although China had a slight discrepancy between the quality and volume of research, it has to be said that China’s performance in this field has been quite outstanding in being able to stand out among numerous countries. In recent years, the change of lifestyle have led to the high incidence of CRC in China, which provided inherent reasons for Chinese scholars to carry on CRA-related research. In order to solve the problem of high incidence of CRC, the persistent exploration of Chinese scholars was also one of the essential factors for China to be at the forefront worldwide in this research field. On the other hand, research in China has grown rapidly over the past few years, which might be related to some achievements of CRA in traditional Chinese medicine; for example, a recent study reported that Berberine, extracted from the Chinese herb, could reduce the risk of recurrence of CRA (24). However, even if many countries had published a large number of papers, collaboration with each other remained limited, such as Japan and China. Therefore, it is highly likely that future novel research breakthroughs in this field will come in the form of active international academic communications.

In addition, the genetic differences caused by racial/ethnic groups are also an important research direction to explore the incidence and prevention of CRA and CRC in the future. In the case of Uruguay and Puerto Rico, two regions in the Americas with a high incidence of CRC, we conducted a search of the literature. Both Uruguay and Puerto Rico are home to large Hispanic populations. Studies have shown that Puerto Ricans living in Florida or California had a higher incidence of CRC than Mexicans living in these states (25). And mortality of CRC in Puerto Rico was higher than in Hispanic American. In terms of genetics, Puerto Ricans have higher levels of African genetic ancestry than most Latino subpopulations, and a higher level of European ancestry than Mexicans (25) Therefore, this phenomenon was most likely due to genetic differences by race. On the other hand, due to the low prevalence of CRC screening, these two regions still had less incidence of CRC than Europe. Studies have shown that CRC was the second most common but the highest mortality rate cancer in Puerto Rico (26). With the improvement of the economic level, the incidence of CRC was the highest among malignant tumors in Uruguay. In summary, it will be one of the future frontiers in the research of CRA and CRC that focusing on the genetic differences caused by racial/ethnic groups, and developing a series of screening strategies based on genetic differences to improve the efficiency of CRC screening.

Among the top 10 institutions in the term of the volume of publications, nine were from the USA and Japan occupied one seat, which was in line with the leading positions of these two countries. This phenomenon might be related to the degree of development and the emphasis on early diagnosis and treatment. Declined CRC incidence has been observed in few affluent countries (27, 28), which mainly benefited from the healthier lifestyle and the establishment of screening programs a decade ago (29). However, as shown in the **Table 2**, Chinese institutions had no place, which indicated that not only the Chinese researchers should improve the quality of research, but also the government should give certain preferential policies to researchers engaged in this field in the future.

Journals and co-cited journals analysis showed that *Cancer epidemiology biomarkers prevention* published the most CRA papers (**Table 3**), while *Gastroenterology* received the largest number of co-cited references (**Table 4**). Moreover, *N Engl J Med* had 12 references in the most cited/co-cited references lists, which was also enough to prove its influence. Therefore, we suggest scholars should take more attentions to these journals to keep track of hotspots and frontiers. Meanwhile, journals at the Q1 JCR division accounted for the majority of the top 10 journals (40%) and co-cited journals (90%). Therefore, we considered that high-quality journals have maintained close contact with the CRA research field. Besides, in recent years, more and more journals have shown great interest in CRA-related papers, which also indicated the vigorous development in this research field (**Figure 5B**).

Highlighting the contributions of influential researchers, such as the authors with many cited and co-cited papers, could help us move along the road and provide further directions and guidelines. In our analysis (**Table 5**, **Figure 6A**), John A Baron from University of North Carolina at Chapel Hill published the highest number of publications in co-authorship analysis; while Sidney J Winawer from Memorial Sloan Kettering Cancer Center had the most co-citations. Meanwhile, John A Baron was also one of the top 10 co-cited authors. We found that he has devoted himself to conducting chemoprevention agents-related research, including aspirin, rofecoxib, calcium and vitamin D, and so on (22, 30, 31). And he has also made substantial efforts in the epidemiology and prevention of CRC (32). Among the 10 leading co-cited authors, Sidney J Winawer was the principal investigator of the landmark in this field (33). He helmed the first study to demonstrate conclusively that removing adenomas reduced the risk of CRC (34); and he was the first to define familial high-risk populations (35), and demonstrate a long-term reduction in incidence and mortality of CRC by removal of adenomatous polyps (36). Meanwhile, we found that papers by two authors, Sandler RS from University of North Carolina at Chapel Hill and Rex DK from Indiana University School of Medicine, also appeared at the top 10 most cited/co-cited references, which also indicated that the remarkable influence of these two authors in this field.

### 4.2 Valuable publications of colorectal adenoma

The valuable references had a great academic impact on the each research field, which was one of the contents we should focus on. The top 10 cited/co-cited references were shown in the **Tables 6**, **8**. There were 18 references in total, excepted for the duplicated references. “Cardiovascular events associated with rofecoxib in a colorectal adenoma chemoprevention trial” has been cited 1,882 times since its publication and was the most frequently quoted research about CRA. This article was published in *N Engl J Med*(IF=91.25) in 2005, and its corresponding author was Bresalier RS from University of Texas M.D. Anderson Cancer Center. This study explored cardiovascular risk of a chemoprevention agent of CRA, rofecoxib. In this randomized, placebo-controlled, double-blind trial, Bresalier RS et al. found that among patients with a history of CRA, the use of rofecoxib, a selective COX-2 inhibitor, was associated with an increased cardiovascular risk, including myocardial infarctions and ischemic cerebrovascular events, and the increased relative risk of thrombotic events was first observed after approximately 18 months of treatment (19). Moreover, two publications appearing both in most cited and most co-cited references lists explored the importance role of prevention in CRA, which were all published in *N Engl J Med.* These two publications were published by Baron JA et al. in 2003 and Coley DA et al. in 2014, respectively. The former found that low-dose aspirin had a moderate chemoprevention effect on CRA (22). The latter found that adenoma detection rate was inversely associated with the risks of interval CRC, advanced-stage interval cancer, and fatal interval cancer (23).

What’s more, these valuable references also focused on exploring the mechanism of CRA. “Comprehensive molecular characterization of human colon and rectal cancer” was published in *Nature* in 2012 (37). This study conducted a genome-scale analysis, analyzing exome sequence, DNA copy number, promoter methylation and messenger RNA and microRNA expression. But most important of all, this team found new gene mutations, and an important role for MYC-directed transcriptional activation and repression, which was undoubtedly of great significance for the early diagnosis and biomarker research of CRA. Another noteworthy reference was “Long-term Expansion of Epithelial Organoids From Human Colon, Adenoma, Adenocarcinoma, and Barrett’s Epithelium”, which was published in *Gastroenterology* in 2011 (38). This reference ranked first with average 148 citations per year. Sato T et al. established colorectal organoids for the first time, which was a foundation work of organoid technology for colorectal tumor. Organoid is a technology enabling researchers to recreate human organs and diseases in a dish and thus holds great promises for many translational applications such as drug discovery and precision medicine. Nowadays, organoids have shown prospects in early biomarker diagnosis, target prediction and drug screening of CRA (39, 40). However, due to the lack of functional vascular system, nervous system and immune system, organoid is still inferior to *in-vivo* models, so it still needs to be explored by researchers. In summary, most of the top 10 most cited/co-cited references focused on prevention and mechanism. Future CRA-related research will be more precise and provide more precise prevention and treatment according to the different characteristics of patients, so as to ultimately achieve the goal of reducing the incidence and mortality of CRC.

### 4.3 Keywords analysis of colorectal adenoma

Keywords analysis included clusters analysis, timeline view analysis and burst analysis, which could show the hotspots and help to understand the direction in an academic field. We found that although the research direction of CRA was relatively extensive, there was a lack of summary and analysis of research hotspots. Therefore, in this paper, we mainly got 4 clusters through the analysis of keywords, so as to help us to extract the main information of hotspots (**Figure 7A**). According to the content of cluster analysis, we summarized and analyzed 4 research hotspots of CRA. Focusing on discussing and explaining these clusters could help identify valuable future directions.

Hotspot 1 mainly focused on mechanism/pathophysiology. Studies showed that CRA developed into CRC, which involved three stages in morphology: hyperproliferative epithelium, adenoma, and carcinoma, which took 10-15 years (41). In this regard, it was particularly important to improve our understanding of the mechanism/pathophysiology of CRA to effectively prevent CRA from developing into CRC. Sequencing research showed that CRA developed into CRC through two main pathways: the chromosomal instability pathway and the microsatellite instability pathway. In these pathways, about 25 common genes played a role in the development of cancers, which included APC, TP53, KRAS, BRAF, etc. The process of CRA into carcinoma mainly included the adenoma-to-carcinoma and the sequence serrated neoplasia pathway (42). The former, which accounted for 85% of colorectal cancerization, was the evolution process of conventional adenoma, and its mutation far exceeded genes that were major drivers. The latter, which accounted for 15% of colorectal cancerization, occurred through an alternative hypermutation pathway, which were mainly subtle mutations that were associated to altering protein products and led to high-frequency microsatellite instability (43, 44). Molecular alternations were mainly hypermethylation of CpG islands, which led to microsatellite instability, rendering the epithelial cells premalignant (45). However, current studies were mostly focused on the molecular signaling pathways of CRA into CRC, few studies were focused on the pathogenesis of CRA, or recurrence after resection. In the future, scholars engaged in CRA-related studies are still needed to further explore these two aspects to treat CRA, so as to achieve the goal of reducing the incidence and mortality of CRC. And with a better understanding of the molecular basis of CRC, the development of diagnostic tests based on more sensitive and specific biomarkers may also provide a breakthrough to the limitations of current screening tests for CRC (46, 47).

Hotspot 2 mainly focused on risk factors and prevention. With the increasing attention to cancer prevention, more and more research on the CRA-related risk factors have been conducted. Currently, risk factors of CRA have been proven to be related to these factors: age (48), physical inactivity, poor diets (49), alcohol drinking, smoking (50), and family history (51). At present, there were many studies on the prevention of CRA, especially chemoprevention agents. The agents that have been confirmed mainly included folic acid (52), vitamin D (53), non-steroidal anti-inflammatory drugs (54), calcium supplement (53), etc., which has been proved that these agents could play a preventive role to a certain extent, but their efficacy was not satisfactory, and had some adverse reactions. This phenomenon has limited broad application of chemoprevention in clinical practice. For example, as shown in the analysis of the most cited references (**Table 6**), the topic of cardiovascular adverse events was also an important research direction. To sum up, there are still huge deficiencies in prevention research of CRA. Therefore, reducing the incidence of CRA from the perspective of prevention is still the research direction that we need to continue to explore in the future. On the other hand, there was a high risk of recurrence after polypectomy, so the research of chemoprevention agents that could effectively prevent recurrence without adverse reactions is also an important research direction in the future. In recent years, as more and more studies have shown that Chinese herbal medicines were safe and effective, some scholars have applied Chinese herbal extracts to the prevention and treatment of recurrence of CRA after polypectomy (24), which provided a new development direction for chemoprevention agents of CRA undoubtedly.

Hotspot 3 mainly focused on colonoscopy screening and treatment. Screening was greatly significant for the early diagnosis and treatment of CRA and CRC. Winawer SJ et al. and Zauber AG et al. published two publications in 1993 and 2012, respectively, showing that colonoscopy screening could reduce the incidence of CRC by 76-90% and the mortality of CRC by 53% through the detection of adenomas (20, 34). These two research established the importance of colonoscopy screening. Colonoscopy and pathology were the gold standards for the diagnosis of CRA and CRC, the final pathway for other screening tests after positive results, and the most appropriate screening method for the population with a family history of CRA and CRC. Besides, with the development of technology, colonoscopy polypectomy, such as endoscopic mucosal resection and endoscopic submucosal dissection, could accurately diagnosis and removal of CRA in time (55). However, colonoscopy screening was an invasive procedure. Therefore, it is still necessary to solve the problems of interval cancer, pain, intestinal damage, and overdiagnosis caused by screening and resection in the future (56). In addition, the differences in frequency of screening for high-risk and low-risk population also needs to be further explored.

Hotspot 4 mainly focused on metabolism. The accumulation of fat in the body could lead to metabolic abnormalities so as to cause metabolic syndrome. Some research have showed that the incidence of CRC was increased in patients with metabolic syndrome, which was an independent risk factors for CRA and CRC (57, 58). On the one hand, metabolic abnormalities related components, such as obesity, insulin resistance, dyslipidemia, diabetes mellitus, would lead to the incidence, development and recurrence of CRA (59). As shown in **Figure 7A**, cluster 4 and cluster 1 had some overlap. On the other hand, the role of abnormal metabolism in the pathogenesis and the regulation of related signaling pathways may provide ideas for the prevention of CRA. Metformin was a drug that could decrease glucose and insulin resistance to treat metabolic abnormalities. In a multicenter, randomized, double-blind, placebo-controlled trial in Japan, low-dose metformin reduced the prevalence and number of metachronous adenomas or polyps after polypectomy (60). In general, these research have proved that the relationship between CRA and metabolic abnormalities to a certain extent, and provided ideas for drug screening to prevent the incidence of CRA from the perspective of treating metabolic abnormalities.

Besides, according to the clustering analysis of the authors (**Figure 6B**), the microbiota was also the hotspot and frontier of CRA. It has been demonstrated by cohort studies that the gut microbiota could directly take part in the progression of CRA and the subsequent development to CRC (61). Japanese scholar Shinichi Yachida et al. was published a cohort study in *Nature Medicine* in 2019 that found microbiome occurred from the very early stages of the development of CRC. 616 participants who underwent colonoscopy to assess taxonomic and functional characteristics of gut microbiota. The result showed that Atopobium parvulum and Actinomyces odontolyticus were significantly increased in multiple polypoid adenomas, which had important implications for early-diagnosis and etiology of CRA (62). Moreover, from a mechanistic perspective, the dysfunction of the gut microbiota would influence metabolic function, active pro-inflammatory and cancer-promoting signaling pathways, which provided basic research support for the incidence of CRA by gut microbiota disorder and the subsequent development to CRC (63).

In summary, this study analyzed the overall condition and trend in the research field of CRA. These objective analysis could provide researchers a general overview of this field, especially to those beginner. Moreover, the result could reveal potential collaboration institutions, countries, partners, and publications, and prospective research hotspots. We exhibited significant milestones of CRA but also provided a better guidance for the future. We also hope that this objective study of bibliometrics could provide ideas for future research in this field.

## 5 Limitations

Though we have analyzed the publications on CRA from 2002 to 2022 as far as possible, there were still some limitations. First, due to the nature of CiteSpace software, only articles and reviews in English and recorded in the WoSCC database were considered in our analysis. Papers in other languages, although few, were not analyzed. Second, since a small number of recently published papers had not yet been indexed, they may not have been included in our analysis. Third, we have not considered the Matthew effect, which might influence the results of bibliometric analysis. These were usually limitations to publication research (64). Nevertheless, the publications based on bibliometric research certainly laid the foundation for researchers to intuitively understand the research hotspots, evolution process, and trends of the CRA.

## 6 Conclusion

At present, this is the first study to analyze the research trends of CRA (2002–2022) by bibliometric methods. The USA published the most publications and the highest H-index, Australia cooperated extensively with other countries, while China performed well and developed rapidly in recent years. The USA had most outstanding institutions and scholars in this field. Updates on the latest research or advances can be found in *Cancer epidemiology biomarkers prevention*, *Gastroenterology*, and *N Engl J Med*. Pathogenesis of CRA, less invasive diagnostic methods, chemoprevention, and screening and risk prediction of CRA including gut microbiome and metabolism as the frontiers of research that should be closely took attention in the future.

## Data availability statement

The original contributions presented in the study are included in the article/supplementary material. Further inquiries can be directed to the corresponding authors.

## Author contributions

XW and HL conceived the study and supervised the manuscript. XL and WZ drafted the manuscript, and prepared the figures and tables. XL and XW extracted all data and performed the bibliometric analyses. SY, QM, CZ, and RC revised the manuscript. All authors contributed to the article and approved the submitted version.
